# Neurosyphilis-Induced Folie À Deux: A Case of Prolonged Psychosis

**DOI:** 10.7759/cureus.46375

**Published:** 2023-10-02

**Authors:** Margarida Alves, Cátia Pinheiro Ramos, Diana Durães, Susana Mendes, António Gamito

**Affiliations:** 1 Department of Psychiatry and Mental Health, Setúbal Hospital Centre, Setúbal, PRT

**Keywords:** neuropsychiatric manifestations, folie à deux, psychosis, syphilis, neurosyphilis

## Abstract

Neurosyphilis presentations can include psychiatric symptoms such as psychosis, mania, depression, or changes in personality. Therefore, it can lead to a misdiagnosis with psychiatric disorders. The diagnosis is particularly difficult in a subset of patients whose psychotic symptoms are developed as a manifestation of the disease later in life.

With the aim to highlight the importance of considering neurosyphilis in the differential diagnosis of late psychosis, it is presented the clinical case of a *folie à deux* from a Portuguese 69-year-old man, who presented symptoms of psychosis in the emergency room, ultimately diagnosed with neurosyphilis.

A selective review of the literature was made using the *Pubmed* database, with “neurosyphilis”, “psychosis”, “syphilis”, and “folie à deux” as keywords. Patient consent was obtained for the use of clinical data.

Neurosyphilis represents the most severe consequence of an untreated syphilis infection and there is a need in the scientific community to establish tools to enhance the precision of diagnosis and treatment.

## Introduction

Syphilis is a sexually transmitted disease caused by the bacterium *Treponema pallidum* that can progress through several stages: primary, secondary, latent, and tertiary syphilis. When untreated individuals may develop tertiary forms like neurosyphilis [1].

Neurosyphilis is a complex bacterial infection that affects the brain or spinal cord and is nowadays less prevalent compared to the pre-penicillin era. Among individuals infected with syphilis only a small proportion of approximately 5% to 10% will develop this particular comorbidity involving the central nervous system and around one-third will display clinical symptoms associated with the disorder [2-4].

In its early stages, neurosyphilis can present as meningitis, meningovascular syphilis, or asymptomatic neurosyphilis. Late-stage neurosyphilis can manifest as paretic neurosyphilis (general paresis) and tabes dorsalis [5].

It is important to note that neurosyphilis can occur at any stage after the initial exposure to syphilis, often manifesting many years or even decades later. However, unlike other bacteria that can infect the cerebrospinal fluid (CSF), the invasion by *Treponema pallidum *does not always result in persistent infection as spontaneous resolution may occur in some cases without an inflammatory response [1].

Neurosyphilis is commonly referred to as "the great imitator" within the scientific community due to its remarkable capacity to mimic various other medical conditions [1]. For example, it may present with a range of psychiatric symptoms including mood changes (depression and mania), psychosis, personality changes, delirium, cognitive impairment, or even dementia [2,4,5].

In this clinical case, we present a *folie à deux* which has at its genesis a neurosyphilis-induced psychosis.

## Case presentation

Mr. A., a 69-year-old Portuguese man without previous psychiatric or medical history, was brought by the police to the emergency psychiatry unit on March 26th, 2023 for observation, after medical suspicion of folie à deux with his partner. Mr. A.’s partner of 10 years had borderline intellectual functioning and was being treated in another psychiatric unit for psychosis.

Mr. A. was previously married, had a daughter, and worked in the restoration field until his forties. By that age he discovered he was gifted - he had the ability to take possession of people’s souls and to heal diseases - and decided to get a divorce, sell everything and start working as a “healer”, helping people with their diseases “balancing patients with the power of the mind”. His presentation was notable for grandiose and mystic delusions, insomnia, and insight impairment.

The patient denied symptomatology compatible with maniform/depressive episodes and a history of substance abuse, so the differential diagnosis of late-onset schizophrenia and unspecified psychosis were considered. The patient was admitted to the psychiatry ward for clinical stabilization and olanzapine 10 mg at bedtime was initiated, with poor response of psychotic symptoms.

Basic psychiatric screening lab tests revealed to be positive for syphilis, with the following results: Rapid plasma reagin (RPR) test positive; Venereal Diseases Research Laboratories (VDRL) test positive with a titer of 1:256; *Treponema pallidum* Hemagglutination Assay (TPHA) positive at 1:20480 dilution and positive Treponemal Enzyme Immunoassay (EIA).

A review of Mr. A.'s past psychiatric and medical history revealed that he had several unprotected sexual relations by the age of 13, during the period he was living with his parents in Angola.

On neurologic examination, the patient presented a left-sided Babinski and Argyl-Robertson pupil. Neuropsychological evaluation indicated graphomotor perseverations and a score of 12/18 was obtained in Frontal Assessment Battery (FAB), the cut-off value.

Due to the long duration of the infection, the lack of dermatological symptoms, and positive serologic results, a late form of syphilis was admitted. The presence of neuropsychiatric symptoms and the alterations at the neurologic exam led to further investigation for possible neurosyphilis.

A lumbar puncture was performed and cerebrospinal fluid (CSF) analysis revealed positivity of TPHA and VDRL at 1:2.

**Figure 1:**
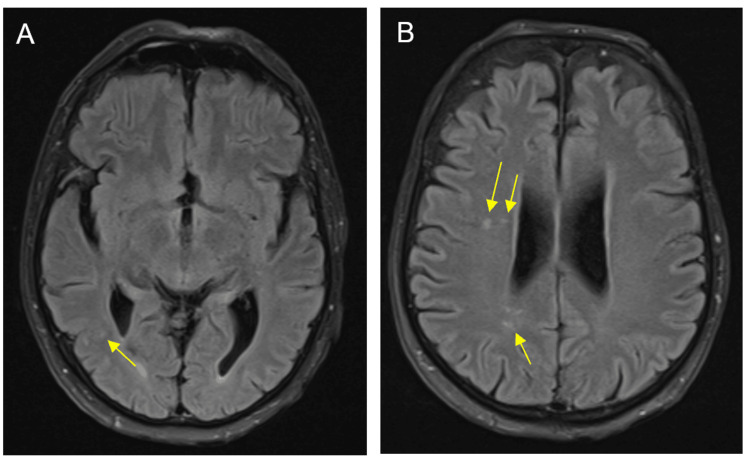
Cranial magnetic resonance Cranial magnetic resonance axial images (A and B) show generalized atrophy and nonspecific foci of hypersignal in the right radial topography and in the right inferior cerebellar hemispherical topography on T2-weighted/FLAIR images (arrows). These later alterations have been previously described in patients with neurosyphilis, however, it cannot be ruled out that these findings could be due to chronic microvascular changes. FLAIR: fluid-attenuated inversion recovery

Cranial computed tomography (CT) revealed discrete atrophy of frontal predominance and nonspecific white matter hypodensities, predominantly in the left frontal region. Cranial magnetic resonance imaging (MRI) showed generalized atrophy, a pattern of mild chronic periventricular microangiopathic leukoencephalopathy and nonspecific foci of hypersignal in the right radial topography and in the right inferior cerebellar hemispherical topography on T2-weighted/fluid-attenuated inversion recovery (FLAIR) images (Figure 1).

Neurosyphilis was diagnosed and, after consultation with the infectious diseases team, the patient was started on four million units of intravenous penicillin G every four hours for 14 days.

During the hospitalization, due to the persistent lack of insight, long-acting injectable paliperidone palmitate 150 mg was initiated. At the time of discharge, there was a sleep regularization and less dynamism of delusions but the lack of insight remained, so that it was necessary to maintain the depot medication on a compulsory basis, according to the Portuguese Mental Health Law. He returned home with his family with the indication for follow-up with his local outpatient psychiatrist and family doctor for further syphilis-related management.

## Discussion

Neurosyphilis can remain asymptomatic with the diagnosis being based on CSF analysis, utilizing non-treponemal tests like the VDRL or RPR tests or treponemal tests such as TPHA [1,6]. On the other hand, the prevalence of neuropsychiatric manifestations in neurosyphilis ranges from 27% to 86%, and psychiatric symptoms are the primary manifestation in about 17% of the patients [7-9]. In one of the largest case series of neurosyphilis in the United Kingdom, psychosis or schizophrenia-like symptoms were the first manifestation in approximately 20% of the cases [10]. These psychiatric symptoms can lead to a misdiagnosis and wrong therapeutic decisions with clinical suspicion playing an important role in the diagnostic process.

Shared psychotic disorder or *folie à deux* is characterized by sharing a delusion among two or more people in a close relationship. *Folie imposee* is a type of shared psychosis in which an individual with psychosis transfers the delusions to another person. Multiple disorders can be at the origin of the psychotic symptoms [11]. Our patient clinical case initially fitted with the diagnosis of late-onset schizophrenia, which has an onset between ages 40-60 years and is characterized by fewer negative and cognitive symptoms, and better premorbid functioning when compared with schizophrenia [12,13]. However, when dealing with an elderly patient who presents with psychosis as the primary symptom, it is essential to consider a wide range of potential causes, including organic diseases like neurosyphilis.

Although it was plausible that the patient could have both late-onset schizophrenia and neurosyphilis, the positive results for syphilis in serum and CSF tests as well as the limited response to antipsychotic treatment indicated that his symptoms were more likely attributed to neurosyphilis. In fact, due to the ability of neurosyphilis to mimic common psychiatric syndromes, some argue for screening all admitted psychiatric patients for syphilis [12].

On brain imaging exams, CT, and MRI, there are no pathognomonic findings that suggest a diagnosis of neurosyphilis. The most common findings are generalized cerebral atrophy, foci-increased signal intensity on T2-weighted images, and a significant association between the presence of frontal lesions and the degree of psychiatric morbidity [14]. In Mr. A.’s case, the exams showed similar findings.

There is limited data on how to treat the psychiatric symptoms associated with neurosyphilis, and psychotic symptoms in particular [15]. Antipsychotic treatment regimens with risperidone, quetiapine, or olanzapine can be found in the literature, but there is currently no consensus [15,16]. In some cases, the psychotic symptoms respond to antibiotic treatment rather than to the antipsychotics [17].

In our clinical case, the patient showed an improvement in psychotic symptoms but didn’t gain insight, therefore it was necessary to continue the treatment with antipsychotic medication, in this case with paliperidone palmitate.

The presence of psychotic symptoms in neurosyphilis is linked to a poor prognosis, as only a portion of individuals experience resolution of psychotic symptoms following intravenous penicillin treatment, with an estimated cure rate of approximately 60%. In cases in which psychotic symptoms are resistant to treatment, the ongoing use of antipsychotic treatment may be necessary. On follow-up, if a patient demonstrates sustained improvement and no longer displays psychotic symptoms, it is recommended to consider discontinuing antipsychotic treatment to minimize potential adverse effects associated with psychotropic medications [2,3,18].

The case of Mr. A. exemplifies the importance of thoroughly considering medical factors when evaluating new-onset psychosis in elderly patients. This is especially crucial when symptoms demonstrate resistance to antipsychotic treatment.

## Conclusions

In conclusion, the pleomorphic psychiatric symptomatology of neurosyphilis presents a challenge in clinical psychiatry. Neurosyphilis, despite its rarity, represents the most severe consequence of an untreated syphilis infection. Fortunately, the prognosis of neurosyphilis has significantly improved in recent years due to the prompt and appropriate administration of antibiotic treatment.

Accurate diagnosis and treatment of neurosyphilis are paramount to prevent irreversible complications. Additionally, there is a pressing need in the scientific community to establish universally accepted diagnostic criteria and tools to enhance the precision and consistency of diagnoses.
